# 

**Published:** 1877-06

**Authors:** 


We have before us a notice of the first annual meeting of the American Dermatological Association, which is to be held at Niagara Falls, on the 4th day of September next.

For the benefit of the profession, and for the advancement of dermatology in America, it is very desirable that this enterprise be a success, and the best way to insure this result is for every one to make manifest the interest they may feel.

The titles of all papers to be read at any annual session shall be forwarded to the Secretary not later than one month before the first day of the session." (By-laws sec. iii.)

The officers of the Association are: James C. White, M.D., President; Louis A. Duhring, M.D., Robert W. Taylor, M.D., Vice-Presidents; L. Duncan Bulkley, M.D., Secretary.

PAMPHLETS RECEIVED.—Tracheotomy in diphtheria. By J. H. Pooley, M.D., Professor of Surgery in Sterling Medical College, Columbus, O. From the June number *Richmond and Louisville Medical Journal.* 1877.

Viburnum Prunifolium (black haw); its Uses in the Treatment of the Diseases of Women. By Edward W. Jenks, M.D., Professor of Medical and Surgical Diseases of Women, and Obstetrics, Detroit Medical College. From vol i. Gynæcological Transactions. 1876.

